# The F-Actin Binding Protein Cortactin Regulates the Dynamics of the Exocytotic Fusion Pore through its SH3 Domain

**DOI:** 10.3389/fncel.2017.00130

**Published:** 2017-05-04

**Authors:** Arlek M. González-Jamett, María J. Guerra, María J. Olivares, Valentina Haro-Acuña, Ximena Baéz-Matus, Jacqueline Vásquez-Navarrete, Fanny Momboisse, Narcisa Martinez-Quiles, Ana M. Cárdenas

**Affiliations:** ^1^Centro Interdisciplinario de Neurociencia de Valparaíso, Facultad de Ciencias, Universidad de ValparaísoValparaíso, Chile; ^2^Departamento de Microbiología (Inmunología), Facultad de Medicina, Universidad Complutense de MadridMadrid, Spain

**Keywords:** exocytosis, fusion pore, actin polymerization, cortactin, N-WASP, neuroendocrine cells, chromaffin cells, catecholamines

## Abstract

Upon cell stimulation, the network of cortical actin filaments is rearranged to facilitate the neurosecretory process. This actin rearrangement includes both disruption of the preexisting actin network and *de novo* actin polymerization. However, the mechanism by which a Ca^2+^ signal elicits the formation of new actin filaments remains uncertain. Cortactin, an actin-binding protein that promotes actin polymerization in synergy with the nucleation promoting factor N-WASP, could play a key role in this mechanism. We addressed this hypothesis by analyzing *de novo* actin polymerization and exocytosis in bovine adrenal chromaffin cells expressing different cortactin or N-WASP domains, or cortactin mutants that fail to interact with proline-rich domain (PRD)-containing proteins, including N-WASP, or to be phosphorylated by Ca^2+^-dependent kinases, such as ERK1/2 and Src. Our results show that the activation of nicotinic receptors in chromaffin cells promotes cortactin translocation to the cell cortex, where it colocalizes with actin filaments. We further found that, in association with PRD-containing proteins, cortactin contributes to the Ca^2+^-dependent formation of F-actin, and regulates fusion pore dynamics and the number of exocytotic events induced by activation of nicotinic receptors. However, whereas the actions of cortactin on the fusion pore dynamics seems to depend on the availability of monomeric actin and its phosphorylation by ERK1/2 and Src kinases, cortactin regulates the extent of exocytosis by a mechanism independent of actin polymerization. Together our findings point out a role for cortactin as a critical modulator of actin filament formation and exocytosis in neuroendocrine cells.

## Introduction

The exocytotic release of neurotransmitters and neuropeptides is a highly regulated process triggered by a rise in cytosolic Ca^2+^, and that relies on the formation of the SNARE complex as well as the Ca^2+^ sensor synaptotagmin (Südhof, 2013). During the fusion process a narrow channel called “fusion pore” is formed. This channel remains open for a variable period of time. Initially, it allows the slow outflow of small molecules (Zhou et al., 1996); later, it can expand leading to the massive release of the vesicular content or alternatively, it can close again resulting in the partial release of the transmitter molecules stored in the vesicle lumen (Alés et al., 1999). Thus, the temporal pattern and amount of transmitter released under different physiological conditions could be tightly regulated by the size and lifetime of the fusion pore (Cárdenas and Marengo, 2016).

In neurosecretory cells, the cortical F-actin meshwork is dynamically remodeled following stimuli that elevate cytosolic Ca^2+^ concentrations (Wollman and Meyer, 2012; Olivares et al., 2014). In this regard, Ca^2+^ concentrations that induce exocytosis promote disruption of the preexisting cortical actin network, as well as formation of new actin filaments (Trifaró et al., 2000; Gasman et al., 2004; Olivares et al., 2014). This actin remodeling modulates different steps of the secretory process, including the formation of active exocytotic sites (Torregrosa-Hetland et al., 2011; Gabel et al., 2015), the motion of the vesicles to those sites (Giner et al., 2005; Papadopulos et al., 2013), and the kinetics (Berberian et al., 2009; Olivares et al., 2014) and mode of exocytosis (Doreian et al., 2008; Wen et al., 2016). Nevertheless, the mechanism by which a Ca^2+^ signal elicits the formation of actin filaments remains poorly understood.

Cortactin is an F-actin binding protein that regulates F-actin dynamics during plasma membrane remodeling processes such as endocytosis (Cao et al., 2010) and formation of growth cones, lamellipodia, podosomes, and invadopodia (Cosen-Binker and Kapus, 2006). Cortactin also coordinates cellular signaling involved in mechanical forces generated by actin polymerization, as observed during axon outgrowth (Kubo et al., 2015). Furthermore, cortactin is phosphorylated on serine and tyrosine residues by Ca^2+^-activated kinases, such as the extracellular signal-regulated protein kinases 1 and 2 (ERK1/2) (Campbell et al., 1999) and Src (Wu et al., 1991). Both types of phosphorylation determine cortactin activity as well as its association with proteins such as the N-WASP (Martinez-Quiles et al., 2004; Tehrani et al., 2007; Kelley et al., 2011), an actin NPF that accumulates at exocytosis sites together with the Arp2/3 nucleation complex and F-actin (Gasman et al., 2004). In ACCs, both ERK1/2 and Src kinases are activated by secretagogues and regulate exocytosis (Allen et al., 1996; Cox and Parsons, 1997; Mendoza et al., 2003). As we recently reported, Src kinases also control Ca^2+^-dependent actin polymerization and fusion pore lifetime in ACCs (Olivares et al., 2014). Thus, as a substrate of these Ca^2+^-activated kinases, cortactin could play a role in coordinating Ca^2+^ signals with F-actin dynamics and exocytosis in neuroendocrine cells. Here, we show that cortactin promotes Ca^2+^-dependent actin polymerization and regulates the last steps of exocytosis in ACCs. The mechanism involves the association of cortactin-SH3 domain to PRD-containing proteins and cortactin phosphorylation at serine and tyrosine residues.

## Materials and Methods

### Reagents

Alexa Fluor 488-G-actin conjugate (Life Technologies, Carlsbad, CA, USA); anti-mouse Cy2 conjugated secondary antibody (Jackson ImmunoResearch, West Grove, PA, USA); ATP (Sigma-Aldrich, St. Louis, MO, USA); bovine serum albumin (Sigma-Aldrich, St. Louis, MO, USA); collagenase B (Roche, Switzerland); 40,6-diamidino-2-phenylindole (Sigma-Aldrich, St. Louis, MO, USA); digitonin (Sigma-Aldrich St. Louis, MO, USA); dimethyl sulfoxide (Merk Company, Germany); 1,1-dimethyl-4-phenyl-pierazinium (RBI Research Biochemicals Natick, MA, USA); Dulbecco’s modified F-12 medium (Gibco BRL, Gaithersburg, MD, USA); EGTA (Sigma-Aldrich, St. Louis, MO, USA); fetal bovine serum (Gibco BRL, Gaithersburg, MD, USA), gentamicin (Gibco/Life Technology, China); glutamatic acid (Sigma-Aldrich, St. Louis, MO, USA); Lucifer yellow (Sigma-Aldrich, St. Louis, MO, USA); latrunculin A (Sigma-Aldrich, St. Louis, MO, USA); monoclonal antibody against cortactin (Abcam, Cambridge, MA, USA); penicillin (OPKO, Chile); Percoll (GE Healthcare, Piscataway, NJ, USA); *p*-formaldehyde (Sigma-Aldrich St. Louis, MO, USA); phalloidin-tetramethyl-rhodamine-(TIRTC)-B-isothiocyanate (Sigma-Aldrich, St. Louis, MO, USA); PIPES (Sigma-Aldrich, St. Louis, MO, USA); poly-L-lysine (Sigma-Aldrich, St. Louis, MO, USA); Triton X-100 (Merck, Germany); U0124 and U0126 (Calbiochem, San Diego, CA, USA).

### Adrenal Chromaffin Cell Culture

Adrenal chromaffin cells were isolated from bovine adrenal glands by collagenase digestion and further separated from other cell types in a Percoll gradient, as previously described (Ardiles et al., 2007). ACCs were then suspended in a 1:1 mixture of Dulbecco’s modified F-12 medium supplemented with 10% of fetal bovine serum, 50 U/ml penicillin and 100 μg/ml gentamicin, cultured at a density of 3 × 10^5^ cells/ml on 0.01% poly-L-lysine treated coverslips and kept at 37°C in a 5% CO_2_ atmosphere.

### Cortactin and N-WASP Constructs

The GST-fusion constructs of the cortactin-N-terminal deletion mutant (amino acids 1–333), cortactin SH3 domain (amino acids 408–546), SH3W525K and N-WASP PRD (amino acids 268–400) were previously described (Martinez-Quiles et al., 2004). The constructs cloned in the PGEX-6P2 vector (Amersham Pharmacia Biotech, Inc., Piscataway, NJ, USA) were expressed in *Escherichia coli* BL21 cells and purified with glutathione-agarose beads (Life Technologies, Carlsbad, CA, USA) using standard protocols. Mouse WT, its mutant W525K and the non-phosphorylatable mutants S405,418A (2A) and Y421,466,482F (3F), all fused to EGFP, were previously described (Nieto-Pelegrin and Martinez-Quiles, 2009).

The Myc-tagged construct of the N-WASP region WGP (amino acids 1–396) encompassing the PRD of N-WASP and lacking the active VCA domain, was previously described by Rohatgi et al. (2000).

### Peptides Microinjection, Transfections, and Pharmacological Treatments

Adrenal chromaffin cells were injected with 5 μM of the GST-fusion peptides of cortactin SH3, SH3W525K or the N-WASP PRD by using an InjectMan system (Eppendorf, Hamburg, Germany) and 0.5 μm-diameter Femtotips (Eppendorf, Hamburg, Germany). All GST-peptides were injected in a buffer solution containing in mM: 139 K^+^- glutamate, 20 PIPES, 5 EGTA, 2 ATP-Mg^2+^, pH 6.6, in the presence of 4% Lucifer yellow, a fluorescent dye that allowed us to identify the injected cells. The injection time was 0.2 s at a pressure of 120 hPa. Then, ACCs were kept in the culture medium at 37°C for 30 min.

For transfections, ACCs were electroporated using an Amaxa Nucleofector 4D (Lonza, Cologne, Germany) according to the manufacturer’s instructions. After transfection, ACCs were cultured in Dulbecco’s modified F-12 medium supplemented with 10% fetal bovine serum and kept at 37°C in a 5% CO_2_ atmosphere, for at least 48 h prior to experimentation.

To study the role of actin polymerization in exocytosis, ACCs were incubated with 2 μM Latrunculin A (LatA), or its vehicle dimethyl sulfoxide (DMSO) 10 min prior to experimentation and throughout the test. To evaluate the role of ERK1/2 signaling in exocytosis, ACCs were incubated with 10 μM of U0126, or its inactive analog U0124, 15 min prior to experimentation and throughout the test.

### Immunofluorescence

For immunocytochemistry, cultured ACCs were kept at a resting condition or stimulated with 50 μM of the nicotinic agonist DMPP for 20 s, fixed with 4% *p*-formaldehyde in PBS (pH 7.4) for 15 min at 4°C and permeabilized with a fixative solution containing 0.1% Triton-X-100. Then, the samples were blocked with 3% bovine serum albumin for 1 h and incubated with a monoclonal antibody against cortactin (1:100) overnight at 4°C and an anti-mouse Cy2 conjugated secondary antibody (1:250) for 1 h at room temperature. Next, after several washes with PBS, samples were incubated with 5 mg/ml DAPI for 15 min. Finally, samples were processed for immunofluorescence and visualized by confocal microscopy (Eclipse Nikon80i; Nikon, Tokyo, Japan), using a 100x oil immersion objective (NA 1.46). Images were captured, using identical exposure settings between compared samples (typically power 50%, gain 7.8 and pinhole 60 μm for the laser 408; power 55%, gain 7.7 and pinhole 60 um for the laser 488, and power 51%, gain 7.5 and pinhole 60 um for the laser 543). All confocal images were acquired at the equatorial plane of the cell and quantifications were done at this focal plane.

For colocalization of cortactin with actin filaments, the samples were additionally incubated with 1 μM of the F-actin-binding toxin phalloidin-tetramethyl-rhodamine-B-isothiocyanate (phalloidin-TRITC) for 15 min after the incubation with the anti-mouse Cy2 conjugated secondary antibody and then visualized by confocal microscopy.

### Actin Polymerization Assay

In order to analyze the formation of new actin filaments, we performed a previously described *de novo* actin polymerization assay (González-Jamett et al., 2013; Olivares et al., 2014). Briefly, cultured ACCs were permeabilized with 20 μM digitonin in a buffer containing in mM: 139 K- glutamate, 20 PIPES, 5 EGTA, 2 ATP-Mg^2+^, pH 6.6, in the presence of 10 μM free Ca^2+^ and 0.3 μM Alexa Fluor 488-G-actin conjugate. Then samples were fixed with PFA, stained with 5 mg/ml DAPI and visualized by confocal microscopy. When specified, the assay was performed in the presence of the different GST-fusion or Myc-tagged peptides at a concentration of 100 nM. All images were captured at the equatorial plane of the cells, using identical exposure settings between compared samples. Confocal images were analyzed and processed using the ImageJ software (NIH, USA).

### Amperometric Recordings

The amperometry set-up consists of an inverted fluorescence microscope (Diaphot-200, Nikon, Japan), equipped with a mercury lamp and a FICT filter set (B-2A, Nikon, Japan), that allows us to identify cells transfected with EGFP constructs or injected with Lucifer yellow. Exocytosis was monitored as previously described (Ardiles et al., 2006) using 5-μm-diameter carbon fibers (Thornel P-55; Amoco, Greenville, SC, USA) and a patch clamp amplifier (EPC-10 USB; HEKA Electronics, Lambrecht, Germany). The amperometric signal was low-pass filtered at 1 KHz and digitalized at 10 KHz with the acquisition software PatchMaster (HEKA Electronics, Lambrecht, Germany). During recordings, cultured ACCs were perfused with a Krebs-Hepes solution (mM: 140 NaCl, 5.9 KCl, 1.2 MgCl_2_, 2 CaCl_2_, 10 Hepes-NaOH, pH 7.4) and exocytosis was induced by a 10 s pressure ejection of 50 μM of DMPP.

### Data Analysis

Confocal images were processed and analyzed using the open access software Image-J (NIH, USA). For determining translocation of cortactin to the cell cortex, we first manually drew the cell outline using the differential interference contrast (DIC) image of each cell to determine the cell area, and measure the TF of each individual cell. We repeated the same process, but drawing 1 μm under the cell periphery, providing the NCF. Finally, the cortical area was obtained by subtracting NCF from TF. This 1 μm annular region corresponds to F-actin-enriched cortex (Doreian et al., 2008; Berberian et al., 2009; Wen et al., 2016). The data are represented as the ratio of CF/TF.

For cortactin/F-actin colocalization analysis the Pearson’s correlation coefficient was measured from each split channel after background subtraction. Fluorescence background was subtracted from a 2x2 pixel region outside the cells using the “ROI background subtraction” plugin of the ImageJ software. Two standard deviations of background from mean fluorescence intensity were typically subtracted.

Amperometric spikes were analyzed using locally written macros for IGOR (Wavemetrics, Lake Oswego, OR, USA) (Segura et al., 2000); this macro can be free downloaded from http://rborges.webs.ull.es/protocols-and-software/. The analysis was restricted to spikes with amplitudes ≥ 10 pA, foot amplitudes ≥ 3 pA and foot durations ≥ 3 ms. Each amperometric parameter was statistically analyzed by taking the median values from individual cells and then averaging these values per treatment group.

Statistical significance was determined utilizing one-way ANOVA followed by an unpaired *t*-test (two-tails).

### Ethics Statement

The present work includes the use of bovine adrenal glands obtained from the Frigorific Don Pedro slaughterhouse (Quilpué, Chile), certified by the Agriculture and Livestock Service of the Chilean Government (certificate number: 04.2.03.0002) and regularly inspected by a Chilean Health Service veterinarian. Transport, processing, and elimination of the samples were carried out in strict accordance with the Article 86 of the Sanitary Regulations of the Chilean Government (Supreme decree N° 977/96).

The protocols described in this article were approved by a Bioethics and Biosafety Committee from the Faculty of Science, University of Valparaíso, directed by Professor Juan Carlos Espinoza, on March 7, 2011.

## Results

### Cortactin Colocalizes with Actin Filament in Stimulated Chromaffin Cells

**FIGURE 1:**
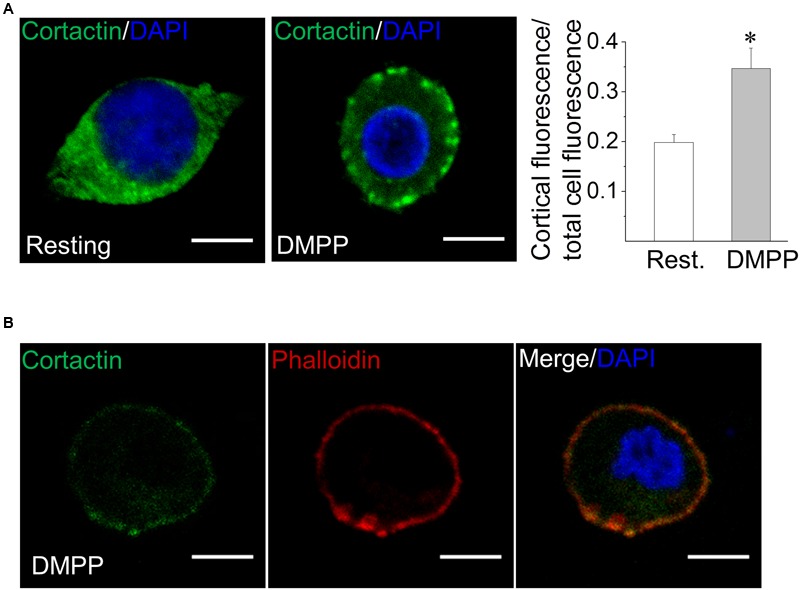
**Cortactin migrates from the cytosol to the cell cortex in response to stimuli that induce exocytosis. (A)** Bovine chromaffin cells under resting condition or stimulated with the nicotinic agonist DMPP (50 μM) were immunolabeled with an anti-cortactin monoclonal antibody (green). Nuclei were stained with DAPI (blue). The relative labeling of cortactin in the cell cortex was quantified by dividing the cortical fluorescence intensity (1 μm under the cell periphery) by the total cell fluorescence intensity. Note that cell stimulation with DMPP significantly increased the cortical localization of cortactin as compared to the resting condition. ^∗^*p* < 0.05 (one-way ANOVA followed by unpaired *t*-test). Scale bar = 10 μm. **(B)** Cultured chromaffin cells were DMPP-stimulated, fixed and co-labeled with an anti-cortactin-directed antibody (green) and the F-actin-binding toxin phalloidin-TRITC (red). Nuclei were stained with DAPI (blue). Then sample were visualized by confocal microscopy. Cortactin/F-actin colocalization was determined by Pearson’s correlation coefficient reaching a value of 0.61 ± 0.05 from five different cultures. Scale bar = 10 μm.

Cortactin is a cytosolic protein that is recruited to the cell cortex to promote cytoskeletal and membrane remodeling (Weed et al., 1998; Lua and Low, 2005; Jacobson et al., 2006). Since the activation of nicotinic acetylcholine receptors triggers exocytosis in chromaffin cells, we analyzed cortactin distribution upon stimulation with the nicotinic agonist DMPP. In resting cells, cortactin is mainly localized in the cytosol, but a fraction of the cortactin staining was found to be localized in the cell cortex (**Figure 1A**). Quantified as a ratio between cortical and total cell cortactin intensity, the fraction of cortactin in the cell cortex in resting condition was 0.2 ± 0.02 (8–10 cells per culture from four different cultures). In cells stimulated with DMPP (50 μM for 20 s), this fraction significantly increased (*p* < 0.05), reaching a value of 0.35 ± 0.04 (8–10 cells per culture from four different cultures). Given the role of cortactin in the remodeling of the cortical actin cytoskeleton, we also examined whether cortactin colocalizes with actin filaments in the DMPP-stimulated condition. Therefore, we stained ACCs with the cortactin-directed antibody and with phalloidin- TRITC-B-isothiocyanate (**Figure 1B**). The degree of colocalization between the two labels was analyzed by using the Pearson’s correlation coefficient. This analysis indicated that cortactin colocalizes with cortical actin filaments (Pearson’s correlation coefficient of 0.61 ± 0.05, from five different cultures).

### Cortactin’s SH3 Domain Enhances the Formation of Actin Filaments in Permeabilized ACCs

We have previously demonstrated that actin polymerization in ACCs is induced by Ca^2+^ concentrations that trigger exocytosis (González-Jamett et al., 2013; Olivares et al., 2014). Therefore, we next determined whether cortactin is involved in this process.

Cortactin can induce actin polymerization through its N-terminal region, which contains Arp2/3 and F-actin binding domains (Uruno et al., 2001). However, this actin-binding protein can also promote the formation of actin filaments by interacting, via its SH3 domain, with PRD-containing proteins such as N-WASP (Martinez-Quiles et al., 2004; Kowalski et al., 2005; Grassart et al., 2010). Therefore, using different regions of cortactin, we explored the implication of these two mechanisms on the cortical F-actin formation induced by Ca^2+^ in ACCs. These experiments were performed in cells permeabilized with 20 μM digitonin, in the presence of 300 nM Alexa-Fluor-488 actin, 2 mM ATP-Mg^2+^ and 10 μM free Ca^2+^. In this condition, a ring of cortical F-actin is formed beneath the plasma membrane (González-Jamett et al., 2013; Olivares et al., 2014).

**FIGURE 2:**
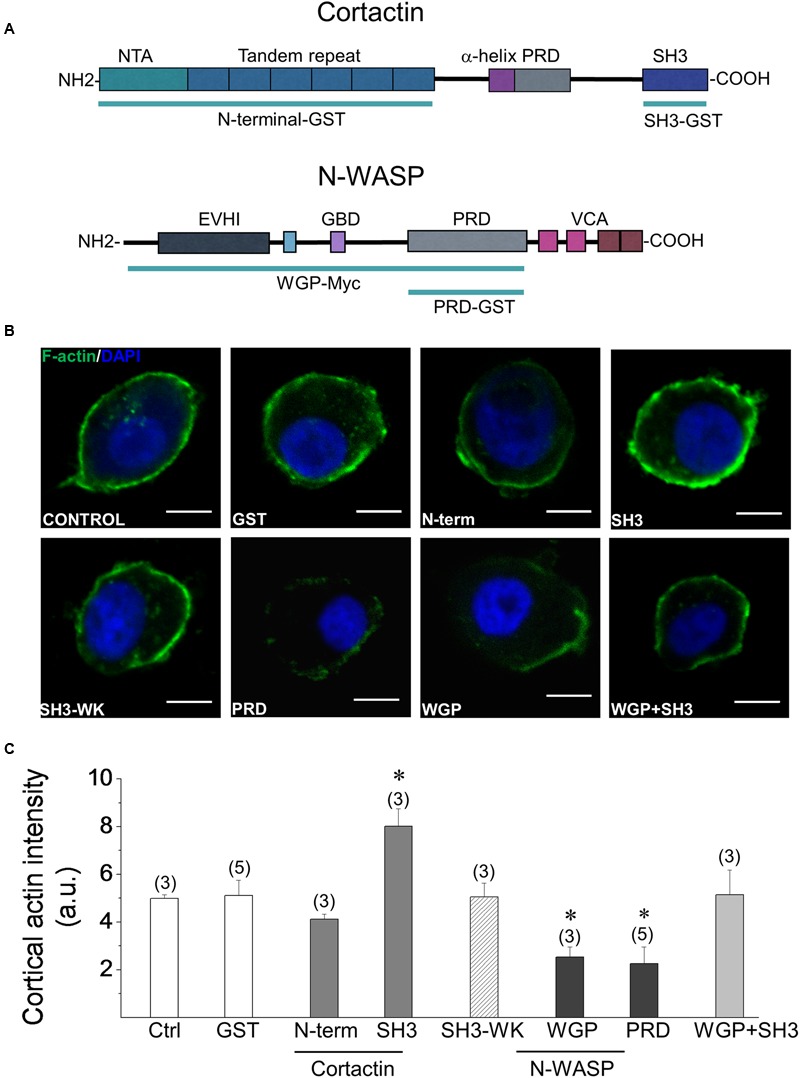
**Effects of the cortactin SH3 domain and the N-WASP PRD on Ca^2+^-induced cortical F-actin formation. (A)** Schematic representation of cortactin and N-WASP primary structures. The cortactin N-terminal region contains the N-terminal acidic domain (NTA), which binds and activates the Arp2/3 complex, and the tandem repeat that binds F-actin. The WGP fragment of N-WASP consists in the protein lacking the VCA region **(B,C)**. F-actin polymerization assay was performed in cells permeabilized with 20 μM digitonin, in the presence of 300 nM Alexa-Fluor-488 actin, 2 mM ATP-Mg^2+^, 10 μM free Ca^2+^ and 100 nM of GST alone or the indicated fusion peptide. Then samples were fixed, stained with DAPI and visualized by confocal microscopy. **(B)** Representative images of F-actin formation. Scale bar = 10 μm. **(C)** The graph corresponds to quantification of the fluorescence intensity of cortical actin filaments 1 μm under the cell periphery. Data are means ± SEM from cells permeabilized in the absence of peptides (control; *n* = 16) or in the presence of GST (*n* = 43), GST-cortactin N-terminal (N-term; *n* = 21), GST-cortactin SH3 (SH3; *n* = 54), GST-cortactin SH3W525K mutant (WK; *n* = 38), Myc-tagged N-WASP WGP (WGP; *n* = 11), GST-N-WASP PRD (PRD; *n* = 33) or GST-cortactin SH3 plus Myc-tagged N-WASP WGP (WGP+SH3; *n* = 25). N corresponds to the number of tested cells per experimental condition from at least three different cultures (numbers over the bars indicate the number of cell cultures). Statistical significance was determined by one-way ANOVA followed by unpaired *t*-test where ^∗^*p* < 0.05 compared with control cells (Ctrl).

We first evaluated the effects of the N-terminal region and SH3 domain of cortactin (see the schematic representation of cortactin domains in **Figure 2A**). Both recombinant peptides were fused to GST. Therefore, their effects were compared with those of GST alone. The SH3 domain was additionally compared with the SH3W525K GST-fusion peptide; this is a mutated version of the cortactin SH3 domain, which possesses reduced ability to bind PRDs (Martinez-Quiles et al., 2004). The SH3W525K peptide did not affect actin polymerization, as compared with cells in the absence of peptides (control) or GST alone (**Figures 2B,C**). Cortactin N-terminal also did not have any significant effect on the formation of cortical F-actin, suggesting that this cortactin region is not critical for F-actin remodeling in ACCs. However, the cortactin SH3-GST-fusion peptide significantly increased the formation of new actin filaments, as compared with cells in the absence of peptides (control), or with cells treated with GST or SH3W525K GST-fusion peptide (*p* < 0.05). Given that the cortactin SH3 domain by itself can bind and activate N-WASP (Martinez-Quiles et al., 2004; Kowalski et al., 2005), we then explored whether the disruption of the association of N-WASP with SH3 domain-containing proteins affects the Ca^2+^-induced formation of actin filaments. Thus, the actin polymerization assay was performed in cells incubated with the N-WASP PRD fused to GST, or with a Myc-tagged peptide of N-WASP WGP (see the schematic representation of N-WASP and these regions in **Figure 2A**). WGP contains the N-WASP PRD but lacks the active VCA region, which contains the motifs that bind actin monomers and the Arp2/3 complex, and promote actin polymerization (Yamaguchi et al., 2000). As shown in **Figures 2B,C**, both N-WASP regions significantly decreased the formation of new cortical actin filaments, suggesting that the interaction of the N-WASP PRD with SH3 domain-containing partners, such as cortactin, regulates F-actin remodeling in ACCs. To address this idea we also evaluated the effect of a co-incubation with equimolar amounts of the cortactin SH3 GST-fusion peptide and the N-WASP WGP. In this condition, the *de novo* formation of actin filaments was not affected (**Figures 2B,C**), suggesting that cortactin/N-WASP association via SH3/PRD interaction plays a key role in the Ca^2+^-induced formation of actin filaments in ACCs.

### Cortactin Regulates Fusion Pore Lifetime through its Association to PRD-Containing Proteins

Given that the SH3 domain of cortactin promotes actin polymerization, whereas the N-WASP PRD inhibits the formation of new actin filaments (**Figures 2B,C**), we subsequently investigated the impact of these actions on exocytosis.

Exocytotic events were induced with 50 μM of the nicotinic agonist DMPP and monitored in real time using amperometry. We quantified the number of amperometric spikes and analyzed the spike charge (Q) that is proportional to the amount of catecholamines released per individual event, and the half-width (t_1/2_) that is proportional to the duration of the exocytotic event (Mosharov and Sulzer, 2005). We also examined the behavior of the early fusion pore, which is observed as a small current that precedes the amperometric spike. We analyzed the duration and amplitude of the foot signal. The duration of the foot signal correlates with the life-time of the initial fusion pore before its enlargement, whereas the foot amplitude is proportional to the flux of catecholamines through the early fusion pore (Fernández-Peruchena et al., 2005). The percentage of spikes with a foot signal was also quantified.

In order to determine the effects of cortactin SH3 domain and N-WASP PRD on exocytosis, ACCs were injected with 5 μM of each GST-fusion peptide. This is a well-suited strategy to determine the acute effects of peptides that interfere with protein functions or associations (González-Jamett et al., 2010).

The effects of the injection of cortactin SH3 domain and N-WASP PRD were compared with the effect of the injection of GST alone. As compared with cells injected only with the injection buffer containing 4% Lucifer yellow, GST alone did not modify the different amperometric parameters (see Supplementary Table S1). Cortactin SH3 effects were additionally compared with the injection of the mutant peptide SH3W525K.

**FIGURE 3:**
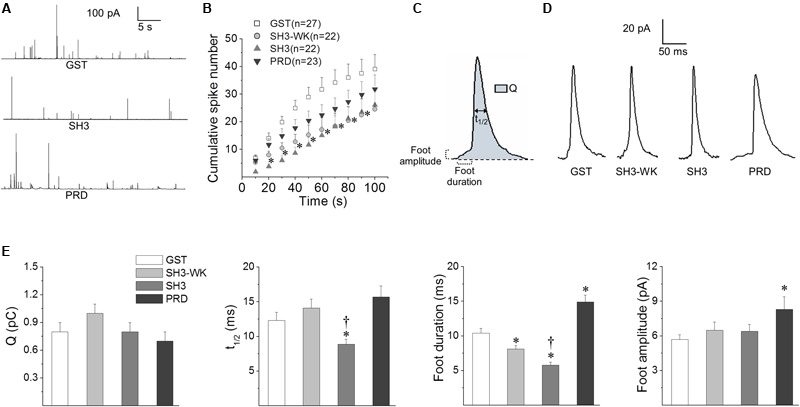
**Effects of the cortactin SH3 domain and the N-WASP PRD on exocytosis.** Chromaffin cells were injected with 5 μM of GST alone, GST-cortactin SH3 (SH3), a mutated version of GST-cortactin SH3 defective in binding PRD (SH3-WK) or GST-N-WASP PRD (PRD). The exocytosis response evoked by a 10 s pulse of 50 μM DMPP was monitored by amperometry 30–45 min after injections. The amperometric recordings lasted 100 s. **(A)** Examples of 40 s amperometric traces from cells injected with GST, SH3 or PRD peptides. **(B)** Cumulative histograms of the number of amperometric spikes from cells injected with GST (white squares), SH3-WK (light-gray circles), SH3 (light-gray triangles) or PRD (dark-gray inverted triangles). Numbers between parentheses indicate the number of cells obtained from at least three different cultures. Notice that both SH3-WK and SH3 significantly reduced the number of spikes between 20 and 80 s. ^∗^*p* < 0.05 compared to GST (one-way ANOVA followed by unpaired *t*-test). **(C)** Scheme of an amperometric spike with the analyzed parameters: quantal size (Q), half width (t_1/2_), foot duration and foot amplitude. **(D)** Examples of amperometric spikes from cells injected with GST, SH3-WK, SH3 or PRD. **(E)** The graphs show mean values of medians determined for single cells of Q, t_1/2_, foot duration and foot amplitude of amperometric spikes from cells injected with GST (white bars), SH3-WK (light-gray bars), SH3 (gray bars) or PRD (dark-gray bars). Data are represented as means ± SEM. Numbers of cells for each condition are the same as indicated in **(B)**. ^∗^*p* < 0.05 compared with GST; ^†^*p* < 0.05 compared with SH3-WK (one-way ANOVA followed by unpaired *t*-test).

Examples of 100 s amperometric recordings induced with 50 μM DMPP in chromaffin cells injected with GST alone, cortactin SH3 or N-WASP PRD GST-fusion peptides are shown in **Figure 3A**. Cumulative histograms of the number of events during the entire recording are shown in **Figure 3B**. As compared with the injection of GST alone, both the SH3W525K and the cortactin SH3 GST-fusion peptide significantly reduced the number of amperometric spikes between 20 and 80 s (*p* < 0.05). However, there were not significant differences between the SH3W525K mutant and cortactin SH3. The N-WASP PRD GST-fusion peptides did not produce any significant effect on the number of spikes (**Figure 3B**).

**Figure 3C** shows a scheme of an amperometric spike with the analyzed parameters. Examples of amperometric spikes from cells injected with GST alone, SH3W525K mutant, cortactin SH3 or N-WASP PRD GST-fusion peptides are shown in **Figure 3D**. The effects of the GST-fusion peptides on the amperometric parameters Q, t_1/2_, foot duration, foot amplitude and percentage of foot signals are show in **Figure 3E** (values are shown in Supplementary Table S1). The injection of the SH3W525K GST-fusion peptide did not affect the amperometric parameters, with the exception of the foot duration that was reduced when compared to GST-injected cells (*p* < 0.05). On the other hand, the injection of the cortactin SH3 GST-fusion peptide did not significantly influence Q, foot amplitude and the percentage of spikes with foot, but it significantly decreased t_1/2_ and foot duration, as compared to either GST alone or the SH3W525K GST-fusion peptide. The N-WASP PRD injection did not affect Q, t_1/2_ or the percentage of spikes with foot signals, but, as compared to GST alone, it significantly increased foot duration and amplitude (**Figure 3E** and Supplementary Table S1). Hence, these results show that, likewise to that observed on the formation of new actin filaments, cortactin SH3 domain and N-WASP PRD have opposite effects on the fusion pore duration.

To further investigate the role of the association of cortactin with PRD-containing proteins, we used another strategy; we expressed the EGFP-tagged FL-W525K. This point mutation severely disrupts the ability of cortactin to bind PRD-containing proteins and also interferes with the F-actin assembly, as shown in HeLa cells (Cantarelli et al., 2006; Nieto-Pelegrin and Martinez-Quiles, 2009). The effects of FL-W525K on exocytosis were compared with the effects of the expression of EGFP-tagged cortactin WT. Therefore, we first determined whether the expression of cortactin WT affects the different amperometric parameters as compared with the expression of the empty vector EGFP. As shown in Supplementary Table S2, cortactin WT did not modify any of the amperometric parameters as compared with the empty vector EGFP.

**FIGURE 4:**
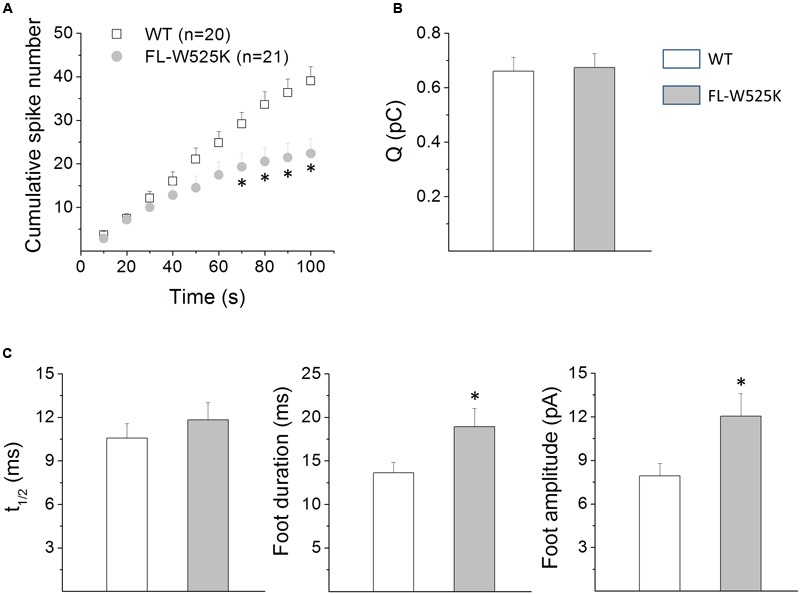
**Effects of the expression of the FL-W525K cortactin mutant on exocytosis in chromaffin cells. (A–C)** Chromaffin cells were transfected with WT, or with the FL-W525K mutant deficient in binding PRD-containing proteins. The exocytosis response evoked by a 10 s pulse of 50 μM DMPP was monitored by amperometry 48 h after transfection. Each amperometric recordings lasted 100 s. **(A)** Cumulative histograms of the number of amperometric spikes during the entire recording in cells transfected with WT cortactin or its FL-W525K mutant. Numbers between parentheses indicate the number of cells analyzed from at least three different cultures. Notice that the number of events is reduced in cells expressing FL-W525K compared to WT during the last 40 s of the recording. ^∗^*p* < 0.05 (unpaired *t*-test). **(B,C)** Graphs represent the mean median values per single cell of the quantal size (Q), half width (t_1/2_) foot duration and foot amplitude of amperometric spikes from cells expressing WT (white bars) or the FL-W525K cortactin mutant (gray bars). Data are means ± SEM. Numbers of cells for each condition are the same as indicated in **(A)**. ^∗^*p* < 0.05 compared with WT (unpaired *t*-test).

**Figure 4** shows the consequence of the expression of FL-W525K on the number of exocytotic events, spike charge (Q), half-width (t_1/2_), foot duration and foot amplitude. As compared with cortactin WT the expression of FL-W525K in ACCs significantly diminished the number of exocytotic events during the last 40 s of the recording (**Figure 4A**). Furthermore, FL-W525K also significantly increased foot duration and amplitude, with no effects on Q, t_1/2_ and the percentage of spikes with foot. The average values are shown in Supplementary Table S3. These results support the importance of the association of cortactin with PRD-containing proteins in exocytosis.

### Effects of the Cortactin SH3 Domain on Actin Polymerization and Exocytosis in the Presence of Latrunculin A

In order to analyze whether the actions of the cortactin SH3 domain on exocytosis depend on actin polymerization, we next further evaluated the effects of the injection of this peptide in the presence of Latrunculin A (LatA). This toxin sequesters monomeric actin and disrupts actin polymerization (Coué et al., 1987). Then, if the effect of cortactin SH3 domain on exocytosis depends on actin polymerization, this peptide would not affect exocytosis in the presence of an agent that sequesters monomeric actin.

**FIGURE 5:**
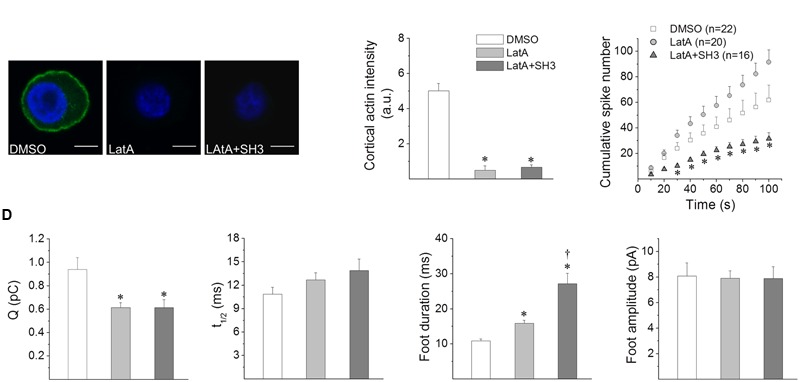
**Effects of cortactin SH3 domain on the exocytosis induced in the presence of latrunculin A. (A,B)** F-actin polymerization assay was performed in cells permeabilized with 20 μM digitonin, in the presence of 300 nM Alexa-Fluor-488 actin, 2 mM ATP-Mg^2+^, 10 μM free Ca^2+^ and the vehicle DMSO, 2 μM latrunculin A (LatA) or 2 μM LatA plus GST-cortactin SH3 (LatA+SH3). Then samples were fixed, stained with DAPI and visualized by confocal microscopy. **(A)** Representative images of F-actin formation. Scale bar = 10 μm. **(B)** The graph corresponds to quantification of the fluorescence intensity of cortical F-actin (1 μm under the cell periphery), where data are means ± SEM of 10–16 cells from three different cultures. ^∗^*p* < 0.05 compared to DMSO (one-way ANOVA followed by unpaired *t*-test). **(C,D)** Cells were incubated for 10 min with 2 μM LatA or DMSO. A group of cells were injected with GST-cortactin SH3 20 min before LatA treatment (LatA+SH3). Exocytosis was induced with 50 μM DMPP and monitored by amperometry. The amperometric recordings lasted 100 s. **(C)** Cumulative histograms of the number of amperometric events from cells treated with DMSO (white squares), LatA (gray circles) or LatA+SH3 (dark-gray triangles). Numbers between parentheses indicate the number of cells analyzed from at least three different cultures. ^∗^*p* < 0.05 compared to DMSO (one-way ANOVA followed by unpaired *t*-test). **(D)** The graphs show mean values of medians determined for single cells of quantal size (Q), half width (t_1/2_), foot duration and foot amplitude of amperometric spikes from cells treated with DMSO (white bars), LatA (gray bars) or LatA+SH3 (dark-gray bars). Data are represented as means ± SEM. Numbers of cells for each condition are the same as indicated in **(C)**. ^∗^*p* < 0.05 compared with DMSO, ^†^*p* < 0.05 compared with LatA (one-way ANOVA followed by unpaired *t*-test).

**Figures 5A,B** show the effects of LatA on the Ca^2+^-dependent actin filament formation; note that, as expected, this agent robustly inhibited actin polymerization. Additionally no F-actin formation was observed in cells treated with both LatA and cortactin SH3 domain (**Figures 5A,B**), indicating that the effects of the cortactin SH3 domain on the formation of new actin filaments depend on the availability of monomeric actin.

**Figure 5C** compares the cumulative histograms of the number of amperometric events induced by DMPP in cells treated with the vehicle DMSO or LatA in non-injected cells, or LatA in cells injected with the cortactin SH3 domain. As compared with DMSO, LatA did not significantly affect the number of exocytotic events. However, in cells injected with the cortactin SH3 domain and treated with LatA, the number of spikes was significantly reduced (**Figure 4C**), indicating that the effects of the cortactin SH3 domain on the number of spikes does not depend on the availability of monomeric actin.

Regarding the effects of LatA on the amperometric parameters, as compared with the vehicle DMSO, this G-actin sequestering agent reduced Q and prolonged foot duration, but it did not affect t_1/2_, foot amplitude and the percentage of foot signals (**Figure 5D** and Supplementary Table S4). LatA induced the same effects in cells concomitantly injected with the cortactin SH3 domain; that is, it reduced Q and prolonged foot duration, without affecting t_1/2_, foot amplitude and the percentage of foot signals. However, the duration of the foot signal was significantly longer in cells injected with the cortactin SH3 domain and treated with LatA, as compared with cells only treated with LatA (**Figure 5D** and Supplementary Table S4).

These results suggest that the actions of the cortactin SH3 domain on the kinetics of single exocytotic events depend on *de novo* actin polymerization. However, this cortactin region appears to regulate the number of exocytotic events through a mechanism that does not involve actin polymerization.

### Serine and Tyrosine Phosphorylation of Cortactin Define Its Action on Exocytosis

**FIGURE 6:**
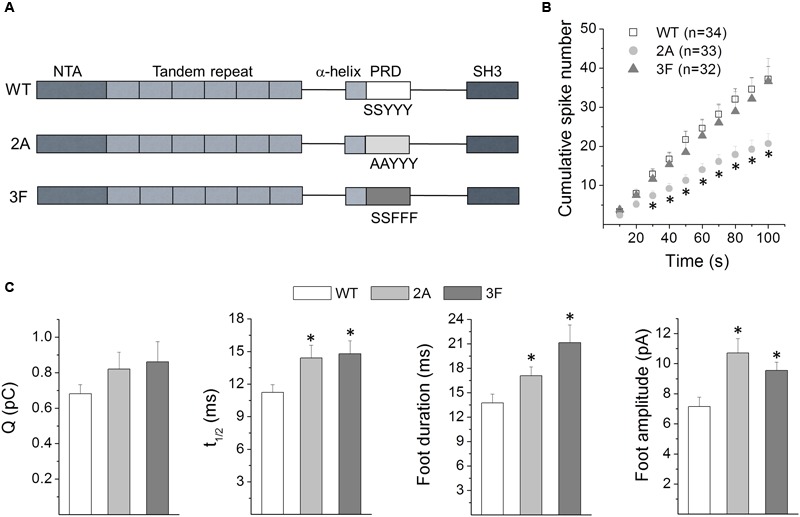
**Effects of serine and tyrosine phosphorylation of cortactin on exocytosis. (A)** Schematic representation of the cortactin primary structure and the location of the mutations used in this study. **(B,C)** Cells were transfected with WT, the ERK1/2 non-phosphorylatable mutant S405,418A (2A) or the Src non-phosphorylatable mutant Y421,466,482F (3F). Exocytosis was induced with 50 μM DMPP and monitored by amperometry 48 h after transfections. Each amperometric recordings lasted 100 s. **(B)** Cumulative histograms of the number of amperometric events from cells expressing WT or the phosphorylation-resistant mutants 2A or 3F. Numbers between parentheses indicate the number of cells obtained from at least three different cultures. Note that in comparison to the WT-expression, the 2A mutant significantly reduced the number of exocytotic events during the last 80 s. ^∗^*p* < 0.05 compared with WT (one-way ANOVA followed by unpaired *t*-test). **(C)** The graphs show mean values of medians determined for individual cells of the quantal size (Q), half width (t_1/2_) foot duration and foot amplitude of the amperometric spikes from cells expressing WT (white bars), 2A (light-gray bars) or 3F (dark-gray bars). Data are represented as means ± SEM. Numbers of cells for each condition are the same as indicated in **(B)**. ^∗^*p* < 0.05 compared with WT (one-way ANOVA followed by unpaired *t*-test).

Cortactin is a substrate of Ca^2+^-activated kinases such as ERK1/2 and Src. ERK1/2, in its active state (pERK1/2), phosphorylates cortactin at the serine residues S405 and S418 (Campbell et al., 1999), while Src phosphorylates cortactin at the tyrosine residues Y421, Y466 and Y482 (Huang et al., 1998). Both types of phosphorylation regulate cortactin association to PRD-containing proteins (Martinez-Quiles et al., 2004; Kelley et al., 2011) as well as its activity following membrane stimulation (Cao et al., 2010; Mezi et al., 2012; Rosales et al., 2012; Vistein and Puthenveedu, 2014). Therefore, we evaluated the effect of the expression of the non-phosphorylatable mutants S405,418A (2A) and Y421,466,482F (3F) on exocytosis in ACCs. Localization of these mutations along the protein is schematized in **Figure 6A**.

The effects of the expression of these cortactin mutants on exocytosis were compared with the expression of cortactin WT. Cumulative histograms of the number of spikes throughout the entire recording period are shown in **Figure 6B**. The expression of the cortactin 3F mutant did not affect the number of amperometric spikes. However, the 2A mutant significantly reduced the number of exocytotic events during the last 80 s, compared with the WT condition. Furthermore, both mutants, 2A and 3F, significantly increased t_1/2_, foot duration and foot amplitude (**Figure 6C**). Mean values of the amperometric parameters Q, t_1/2_, foot duration, foot amplitude and percentage of foot signals for each condition are shown in Supplementary Table S5.

Together these results indicate that the cortactin phosphorylation status influences the features of the exocytotic events in neuroendocrine chromaffin cells.

### Effects of the Inhibition of ERK1/2 Signaling on Exocytosis

Given that the cortactin non-phosphorylatable mutant 2A (**Figure 6**) significantly impacts exocytosis affecting the number of exocytotic events and the fusion pore properties, we analyzed whether the inhibition of the ERK1/2 signaling affects similarly the exocytosis. To do so, we evaluated the effect of U0126, an inhibitor of the MEK. This is a threonine and tyrosine recognition kinase that phosphorylates and activates ERK1/2 (Payne et al., 1991). Therefore, ACCs were incubated with 10 μM of U0126, or its inactive analog U0124, 15 min prior to experimentation and throughout the test. In these conditions, U0126 efficiently inhibits ERK1/2 phosphorylation (Evans et al., 2002; Mendoza et al., 2003).

**Table 1** shows the values of the different amperometric parameters of ACCs treated with U0124 or U0126. As compared with the inactive analog U0124, the inhibitor of the ERK1/2 signaling U0126 reduced the number of exocytotic events, prolonged foot duration and decreased foot amplitude. Q and t_1/2_ were not affected. Then, these results indicate that the ERK1/2 signaling also prominently regulates exocytosis.

**Table 1:** Effects of ERK1/2 signaling inhibition on exocytosis.

	U0124	U0126
Number of events	27.6 ± 4.0	16.4 ± 3.5^∗^
Q (pC)	0.6 ± 0.06	0.5 ± 0.06
t_1/2_ (ms)	12.7 ± 1.4	13.0 ± 1.5
Foot duration (ms)	11.7 ± 0.9	19.1 ± 1.7^∗^
Foot amplitude (pA)	8.0 ± 0.9	5.8 ± 0.5^∗^
Percentage of feet	34.6 ± 3.5	33.7 ± 3.1
Number of cells	22	22

*Chromaffin cells were treated with the ERK1/2 signaling inhibitor U0126 or its inactive analog U0124 15 min before and during the induction of exocytosis with 50 μM DMPP. Data are means ± SEM of median value determined for each cell. ^∗^*p* < 0.05 compared with cells treated with U0124 (unpaired *t*-test).*

## Discussion

The cortical actin network is dynamically reorganized in stimulated neurosecretory cells to allow the secretory process to proceed (Meunier and Gutiérrez, 2016). In this regard, the cortical actin filaments reportedly form polygonal cavities that trap secretory vesicles in the cell cortex and direct them to docking sites (Giner et al., 2005; Papadopulos et al., 2013, 2015), organize active exocytotic sites (Torregrosa-Hetland et al., 2011; Gabel et al., 2015) and regulate the later stages of the fusion process (Berberian et al., 2009; Olivares et al., 2014; Wen et al., 2016). All these functions depend on the inherent plasticity of the actin cytoskeleton and an armamentarium of molecules that control F-actin rearrangement. One of those molecules is cortactin, an actin NPF that binds F-actin and the Arp2/3 complex (Kirkbride et al., 2011), as well as other NPFs such as N-WASP and WAVE2 (Martinez-Quiles et al., 2004; Han et al., 2014), the actin-depolymerizing factor cofilin (Oser et al., 2009) and the GTPase dynamin-2, which is involved in actin filament remodeling (Mooren et al., 2009), among others. In this regard, cortactin can regulate F-actin’s dynamics by using different mechanisms, whose activation varies depending on the cellular process being executed (Kelley et al., 2011; Kirkbride et al., 2011; Helgeson et al., 2014). By analyzing the formation of new actin filaments in permeabilized chromaffin cells and monitoring single exocytotic events with amperometry, we demonstrate here that in ACCs cortactin contributes to the Ca^2+^-dependent formation of new actin filaments and controls the later stages of exocytosis by a mechanism that depends on its association to PRD-containing proteins and its phosphorylation on serine and tyrosine residues by Ca^2+^-dependent kinases.

### Cortactin Translocates to the Cell Cortex upon Activation of Nicotinic Receptors

As shown **Figure 1** cortactin partially translocates to the cell cortex upon cell stimulation in ACCs, where it colocalizes with actin filaments. Cortactin translocation has been observed in different types of cells (Weed et al., 1998; Cantarelli et al., 2000; Tilghman and Hoover, 2002), and reportedly depends on the activation of the Rho GTPase Rac (Weed et al., 1998). This Rho GTPase is activated by cytosolic Ca^2+^ increments (Price et al., 2003) and involved in Ca^2+^-dependent exocytosis in PC12 cells (Komuro et al., 1996; Momboisse et al., 2009). More recently, it has been observed that activation of the protein kinase C promotes cortactin translocation to the cell cortex in a way dependent on the Kelch-like ECH-associated protein 1 (Keap1), a protein involved in the response to oxidative stress (Ito et al., 2015). Importantly, cortactin translocation is required for the initiation of F-actin-dependent processes in different cell types (Weed et al., 2000; Webb et al., 2006).

### Cortactin Controls the Formation of New Actin Filaments via Its SH3 Domain

Cortactin controls actin polymerization in different cellular systems by acting via a mechanism mediated by its N-terminal region, or alternatively through its SH3 domain (Lua and Low, 2005). The N-terminal region of cortactin directly binds to F-actin and activates the nucleation complex Arp2/3 (Uruno et al., 2001), although, as compared with N-WASP, cortactin is a weak NPF (Uruno et al., 2001; Weaver et al., 2001). Additionally, cortactin can act by enhancing N-WASP activity via a SH3/PRD interaction (Martinez-Quiles et al., 2004). Our data suggest that in ACCs cortactin regulates Ca^2+^-dependent actin polymerization mainly through the second mechanism, wherein cortactin appears to enhance N-WASP activity in a manner dependent on their SH3/PRD association. This idea is supported by the following facts: (1) the cortactin’s SH3 domain enhances the formation of actin filaments, (2) both the N-WASP PRD and WGP, an N-WASP region harboring the PRD, reduced the cortical F-actin formation and (3) equimolar amounts of WGP and the cortactin SH3 domain did not affect actin polymerization (**Figure 2**). Nevertheless, we cannot rule out that cortactin could also control actin dynamics by using other mechanisms. In this regard, cortactin could increase, via a SH3/PRD interaction, the intrinsic GTPase activity of dynamin-2 (Mooren et al., 2009), a protein that regulates F-actin dynamics in different types of cells, including ACCs (González-Jamett et al., 2014). Cortactin also associates via a SH3/PRD interaction to WAVE2 (Han et al., 2014), a ubiquitous member of the WAVE that activates the Arp2/3 complex to induce actin polymerization (Nakanishi et al., 2007). Interestingly, all these proteins can work cooperatively in cytoskeleton and membrane remodeling processes, as has been described for the cortactin-N-WASP-dynamin complex (Buccione et al., 2004).

### Cortactin Regulates Fusion Pore Properties via its SH3 Domain

Recent findings have highlighted the role of actin filaments on the later stages of exocytosis. In this regard, Wu and collaborators recently proposed that F-actin favors the membrane tension required to pull the Ω shape membrane profile formed by the vesicle during exocytosis (Wen et al., 2016).

Given that both the cortactin SH3 domain and N-WASP PRD influence the Ca^2+^-induced actin polymerization in ACCs, we determined the impact of these protein domains on exocytosis. As shown in **Figure 3**, the injection of the cortactin SH3 domain reduced t_1/2_ and foot duration, indicating that this cortactin domain, which reportedly activates N-WASP (Martinez-Quiles et al., 2004; Kowalski et al., 2005), speeds up the fusion process. Conversely, the N-WASP PRD lengthened the duration of the foot signal, and increased its amplitude. The expression of the FL-W525K, which reportedly disrupts the ability of cortactin to bind PRD-containing proteins (Cantarelli et al., 2006; Nieto-Pelegrin and Martinez-Quiles, 2009), also increased both duration and amplitude of the foot signals (**Figure 4**). Then, these data suggest that the association of cortactin with PRD-containing partners regulates the kinetics, as well as the conductance of the initial fusion pore. The flux of catecholamines through the initial fusion pore depends on structural features, such as pore diameter and length (Fang et al., 2008). However, vesicle size and intravesicular free catecholamine concentrations could also affect the characteristics of the amperometric foot (Sombers et al., 2004; Amatore et al., 2005). Since amperometry does not directly measure the fusion pore conductance, other types of techniques such as cell-attached capacitance measurements could provide us with additional information about the mechanism by which cortactin regulates the initial pore features.

### Cortactin Regulates the Number of Exocytotic Events

The amount of exocytotic events in neuroendocrine cells depends on the size of releasable pool of vesicles, as well as on vesicle transport, docking, priming, and fusion (Becherer and Rettig, 2006).

As shown in **Figure 4A**, the expression of the FL-W525K, as compared with cortactin WT reduced the number of amperometric spikes. Since this effect is also observed with the cortactin SH3 domain, we could infer that both this peptide and the mutation in the full-length protein interrupt the association of endogenous cortactin with a partner that regulates the amount of exocytosis. However, the mutant peptide SH3W525K also significantly reduced the number of amperometric spikes (**Figure 3B**). A possible explanation for these findings is that the W525K mutation in the full-length cortactin effectively disrupts the association of cortactin to a given PRD-containing domain partner, but this mutation in the GST-peptide is less effective in disrupting that interaction. These differences could be a consequence of phosphorylation or other types of post-transcriptional modifications in the full-length protein that modify its association to a given PRD-containing partner. For instance, cortactin phosphorylation by pERK1/2 enhances its association to N-WASP (Martinez-Quiles et al., 2004), whereas its phosphorylation by Src kinase appears to favor its interaction with dynamin-2 (Cao et al., 2010). Another explanation is that the cortactin SH3 domain could display non-conventional interactions, as observed with other SH3 domains (Jia et al., 2005; Sriram and Birge, 2012). For instance, cortactin interacts with the myosin light chain kinase (MLCK), in a cortactin SH3 domain dependent manner (Dudek et al., 2002, 2004); however, the substitution of proline residues by alanines in the full length MLCK does not alter its association to cortactin (Belvitch et al., 2014). Interestingly, inhibition of MLCK in ACCs hinders Ca^2+^-induced catecholamine release and ATP-dependent vesicle priming and vesicle transport (Kumakura et al., 1994; Neco et al., 2002). Then, MLCK could be a cortactin partner that regulates the amount of exocytosis. Nevertheless, it is important to keep in mind that SH3 domains can exhibit high versatility and promiscuity (Agrawal and Kishan, 2002; Li, 2005), and that the association to PRD-containing partners is regulated by post-transcriptional modifications (Lua and Low, 2005; Sriram and Birge, 2012). These properties allow them to be involved in different types of cellular processes.

### Do the Actions of the Cortactin SH3 Domain on Exocytosis Depend on Actin Polymerization?

Although the effects of the cortactin SH3 domain and N-WASP PRD on the Ca^2+^-induced actin polymerization correlates well with the effects of these peptides on fusion pore dynamics, we cannot rule out that cortactin regulates exocytosis through a mechanism independent of F-actin remodeling. Therefore, to better understand this mechanism, we analyzed the effects of the SH3 domain of cortactin in the presence of LatA, a toxin that sequesters monomeric actin and inhibits actin polymerization. As previously observed by other authors (Berberian et al., 2009), LatA increased the fusion pore duration (**Figure 5D**). This toxin also decreased quantal size (**Figure 5D**), probably as a consequence of an incomplete fusion. In fact, LatA reduces the membrane tension required for vesicle merging into the plasma membrane in ACCs (Wen et al., 2016).

In the presence of LatA, the cortactin SH3 domain was unable to promote actin polymerization (**Figure 5A**), and the effects of LatA on the kinetics of exocytosis predominate over the effects of the cortactin SH3 domain. Indeed, cortactin did not reduce t_1/2_ and foot duration (**Figure 5D**), as observed in cells injected with this peptide but not treated with LatA (**Figure 3E**). Furthermore, foot durations were longer in cells injected with cortactin SH3 domain and then treated with LatA, as compared with non-injected cells treated LatA. A possible explanation for this finding is that LatA might unmask actions of the cortactin SH3 domain, such as its association to partners that regulate fusion pore dilation in a way independent of actin polymerization. That could be the case of MLCK, whose substrate myosin light chain regulates fusion pore expansion in ACCs (Neco et al., 2008).

On the other hand, the cortactin SH3 domain was still able of reducing the number of exocytotic events in the presence of LatA (**Figure 5C**), suggesting that this cortactin action occurs by a mechanism independent of actin polymerization. As we discussed above, MLCK could be a cortactin partner that regulates the amount of exocytosis.

### Serine and Tyrosine Phosphorylation of Cortactin Regulates Fusion Pore Lifetime

As aforementioned cortactin phosphorylation at the serine residues 405 and 418 and at the tyrosine residues 421, 466 and 482, by pERK1/2 and Src tyrosine kinases respectively, regulates cortactin activity and its association with partners (Martinez-Quiles et al., 2004; Tehrani et al., 2007; Kelley et al., 2011). Whereas cortactin phosphorylation by pERK1/2 directly promotes N-WASP activation via a SH3/PRD interaction (Martinez-Quiles et al., 2004), phosphorylation of cortactin by Src activates N-WASP through recruitment of the adaptor protein Nck1, by a mechanism that does not involve the SH3 domain of cortactin (Tehrani et al., 2007; Oser et al., 2009). In this latter mechanism, N-WASP is activated by the SH3 domains of Nck (Rohatgi et al., 2001; Ditlev et al., 2012). Therefore, we expected that the ERK1/2 non-phosphorylatable mutant, but not necessarily the Src non-phosphorylatable mutant, displays effects comparable to those produced by the disruption of cortactin/N-WASP association. Effectively, the 2A mutant prolonged the duration of the initial fusion pore and increased the amplitude of the foot signal, similarly to that observed with the injection of the N-WASP PRD or with the expression of the FL-W525K. Interestingly, the same effects also were observed in cells expressing the 3F mutant, indicating that this type of cortactin phosphorylation can also regulate fusion pore dynamics. Given that cortactin can be simultaneously phosphorylated by both pERK1/2 and tyrosine kinases (Kelley et al., 2011), it is probable that both types of phosphorylation contribute together to regulate fusion pore expansion in ACCs. On the other hand, and contrary to that observed with the SH3 domain of cortactin, both non-phosphorylatable mutants 2A and 3F significantly increased t_1/2_, suggesting that cortactin is involved in the regulation of the duration of the release events.

The effects of the cortactin non-phosphorylatable mutant 3F on t_1/2_ and foot duration correlate well with the effects of the pharmacological inhibition of Src kinases on those parameters (Olivares et al., 2014). On the other hand, both the expression of the non-phosphorylatable mutant 2A and the ERK1/2 signaling inhibitor U0126 prolonged foot duration. However, they have opposite effects on foot amplitude; whereas the 2A mutant prolongs foot amplitude, U0126 decreases it (**Figure 5C** and **Table 1**). pERK1/2 also phosphorylates the MLCK (Klemke et al., 1997), and inhibition of ERK1/2 signaling decreases the function of MLCK, as well as the phosphorylation of its substrate myosin light chain (Klemke et al., 1997). Expression of a non-phosphorylatable mutant of myosin II regulatory light chain in ACCs hinders the fusion pore expansion, limiting the release of catecholamines through the initial fusion pore (Neco et al., 2008). Therefore, the effects of U0126 on fusion pore conductance could be a consequence of an inhibition of MLCK.

Both the expression of the phosphorylatable mutant 2A and the cell treatment with the ERK1/2 signaling inhibitor U0126 reduced the number of exocytotic events, suggesting that cortactin phosphorylation by pERK1/2 influences the amount of exocytosis. As discussed above, MLCK is a cortactin partner involved in vesicle transport and priming (Kumakura et al., 1994; Neco et al., 2002). Furthermore, and as aforementioned, MLCK is also a substrate of ERK1/2 (Klemke et al., 1997). Then these findings point out to ERK1/2, MLCK and cortactin as regulators of the amount of exocytosis.

## Conclusion

Together, the present findings point out a role of cortactin as a modulator of Ca^2+^ regulated exocytosis. Cortactin actions on exocytosis depend on the interaction of its SH3 domain with PRD-containing partners, such as N-WASP and MLCK. Whereas the association of cortactin with N-WASP appears to be critical for the Ca^2+^-induced actin filament formation and fusion pore expansion, a different mechanism seems to determine the role of cortactin in the extent of exocytosis. These cortactin actions are regulated by its phosphorylation at serine and tyrosine residues by ERK1/2 and Src kinases, respectively. These new mechanisms are clearly relevant for the tight regulation of transmitter release in neuroendocrine cells.

## Author Contributions

AG-J: designed and performed experiments, performed statistical analysis, interpreted results, helped draft parts of the manuscript and critically revised the manuscript; MG, MO, VH-A, XB-M, and JV-N: performed experiments and analyzed data; FM: analyzed and interpreted data and critically revised the manuscript; NM-Q: designed constructs, interpreted results and critically revised the manuscript. AC: conceived the study, designed experiments, interpreted results, performed statistical analysis and drafted the manuscript. All authors red and approved the final manuscript.

## Conflict of Interest Statement

The authors declare that the research was conducted in the absence of any commercial or financial relationships that could be construed as a potential conflict of interest.

## Abbreviations

ACCs: adrenal chromaffin cells
2A: ERK1/2 non-phosphorylatable cortactin mutant S405,418A
CF: cortical fluorescence
DAPI: 40,6-diamidino-2-phenylindole
DMPP: 1,1-dimethyl-4-phenyl-pierazinium
ERK1/2: extracellular signal-regulated protein kinases 1 and 2
3F: Src non-phosphorylatable cortactin mutant Y421,466,482F
FL-W525K: full-length cortactin mutant W525K
LatA: latrunculin A
MEK: MAP and ERK kinase
NCF: cell fluorescence with no cell cortex
NPF: nucleation promoting factor
NTA: N-terminal acidic domain
N-WASP: neural Wiskott-Aldrich syndrome
PBS: phosphate-buffered saline; active or phosphorylated ERK1/2 (pERK1/2)
PFA: *p*-formaldehyde
PRD: proline-rich domain
Q: spike charge
t_1/2_: half-width
TF: total fluorescence intensity
TRITC: tetramethyl-rhodamine-B-isothiocyanate
VCA: verprolin-cofilin homology-acidic
WAVE: WASP-family verprolin-homologous protein
WGP: Wiskott-Aldrich GTPase proline-rich domain
WT: cortactin wild-type
