# Supplementary material for: The F-Actin Binding Protein Cortactin Regulates the Dynamics of the Exocytotic Fusion Pore through its SH3 Domain
Source: Front Cell Neurosci. 2017 May 4;11:130. doi: 10.3389/fncel.2017.00130 (PMC5415606; doi:10.3389/fncel.2017.00130)
Supplement: Supplementary file 1 [file Table_1.docx]

**Table S1**: *Amperometric parameters of exocytotic events in cells injected with cortactin and N-WASP domains*. Chromaffin cells were injected with the injection buffer containing Lucifer yellow (LY), GST alone (GST), GST-cortactin SH3W525K mutant (SH3-WK), GST-cortactin SH3 (SH3) or GST-N-WASP PRD (PRD). Exocytosis was induced with 50 µM DMPP and monitored by amperometry 30 min after injections. Data are means ± SEM of median value determined for each cell. ^*^p<0.05 compared with cells injected with GST; †p<0.05 compared with SH3-WK (ANOVA followed by unpaired t-test).

|  | LY | GST | SH3-WK | SH3 | PRD |
| --- | --- | --- | --- | --- | --- |
| Number of events | 35.6 ± 4.1 | 39.0 ± 5.3 | 24.6 ± 6.9 | 26.0 ± 5.1 | 31.8 ± 5.2 |
| Q (pC) | 0.7 ± 0.1 | 0.8 ± 0.1 | 1.0 ± 0.1 | 0.8 ± 0.1 | 0.7 ± 0.05 |
| t_1/2_ (ms) | 10.8 ± 0.7 | 12.3 ± 1.2 | 14.1 ± 1.3 | 8.9 ± 0.7*^†^ | 15.7 ± 1.6 |
| Foot duration (ms) | 10.4 ± 0.4 | 10.4 ± 0.7 | 8.1 ± 0.5* | 5.8 ± 0.4*^†^ | 14.9 ± 1.0* |
| Foot amplitude (pA) | 6.5 ± 0.4 | 5.7 ± 0.4 | 6.5 ± 0.7 | 6.4 ± 0.6 | 8.3 ± 1.1* |
| Percentage of feet | 43.5 ± 3.3 | 38.1 ± 4.5 | 49.1 ± 5.0 | 46.5 ± 4.9 | 39.8 ± 3.9 |
| Number of cells | 28 | 27 | 22 | 22 | 23 |
