# Supplementary material for: The F-Actin Binding Protein Cortactin Regulates the Dynamics of the Exocytotic Fusion Pore through its SH3 Domain
Source: Front Cell Neurosci. 2017 May 4;11:130. doi: 10.3389/fncel.2017.00130 (PMC5415606; doi:10.3389/fncel.2017.00130)
Supplement: Supplementary file 2 [file Table_2.DOCX]

**Table S2**: *Amperometric parameters in cells expressing cortactin wild type cortactin*. Chromaffin cells were transfected with the empty vector (EGFP) or cortactin wild-type (WT). 48 h later, exocytosis was induced with 50 µM DMPP and monitored by amperometry. Data are means ± SEM of median value determined for each cell. *p<0.05 compared with cells transfected with EGPF (unpaired t-test).

|  | EGFP | WT |
| --- | --- | --- |
| Number of events | 32.8 ± 4.8 | 37.2 ± 3.2 |
| Q (pC) | 0.8 ± 0.1 | 0.7 ± 0.1 |
| t_1/2_ (ms) | 9.7 ± 0.5 | 11.4 ± 0.7 |
| Foot duration (ms) | 12.0 ± 0.9 | 13.8 ± 1.1 |
| Foot amplitude (pA) | 6.8 ± 0.6 | 7.2 ± 0.6 |
| Percentage of feet | 52.1 ± 3.0 | 43.4± 3.4 |
| Number of cells | 40 | 34 |
