# Supplementary material for: The F-Actin Binding Protein Cortactin Regulates the Dynamics of the Exocytotic Fusion Pore through its SH3 Domain
Source: Front Cell Neurosci. 2017 May 4;11:130. doi: 10.3389/fncel.2017.00130 (PMC5415606; doi:10.3389/fncel.2017.00130)
Supplement: Supplementary file 3 [file Table_3.DOCX]

**Table S3**: *Amperometric parameters of exocytotic in cells expressing the cortactin mutant W525K*. Chromaffin cells were transfected with cortactin wild-type (WT) or full-length cortactin mutant W525K (FL-W525K). Exocytosis was induced with 50 µM DMPP and monitored by amperometry 48 h after transfections. Data are means ± SEM of median value determined for each cell. *p<0.05 compared with cells transfected with cortactin WT (unpaired t-test).

|  | WT | FL-W525K |
| --- | --- | --- |
| Number of events | 39.1 ± 3.3 | 22.4 ± 3.4* |
| Q (pC) | 0.7 ± 0.1 | 0.7 ± 0.1 |
| t_1/2_ (ms) | 10.6 ± 1.0 | 11.8 ± 1.2 |
| Foot duration (ms) | 13.6 ± 1.2 | 18.9 ± 2.1* |
| Foot amplitude (pA) | 7.9 ± 0.8 | 12.0 ± 1.5* |
| Percentage of feet | 42.3 ± 2.6 | 44.3 ± 3.0 |
| Number of cells | 20 | 21 |
