# Supplementary material for: The F-Actin Binding Protein Cortactin Regulates the Dynamics of the Exocytotic Fusion Pore through its SH3 Domain
Source: Front Cell Neurosci. 2017 May 4;11:130. doi: 10.3389/fncel.2017.00130 (PMC5415606; doi:10.3389/fncel.2017.00130)
Supplement: Supplementary file 4 [file Table_4.docx]

**Table S4**: ***Amperometric parameters of exocytotic events induced by 50 μM DMPP in cells treated with latrunculin A*.** Exocytosis was induced with 50 μM DMPP and monitored by amperometry. Cells were incubated with 2 μM latrunculin A (LatA) or its vehicle 10 min before experiments. A group of cells were injected with SH3 cortactin (SH3) 20 min before the treatment with LatA. Data are means ± SEM. *p<0.05 compared with cells treated with DMSO, ^†^p0.05 compared with cells treated with LatA (Kruskal-Wallis test and Mann-Whitney post-hoc tests).

|  | DMSO | LatA | LatA + SH3 |
| --- | --- | --- | --- |
| Number of events | 61.8 ± 11.7 | 91.5 ± 9.7 | 31.6 ± 4.7*^†^ |
| Q (pC) | 0.94 ± 0.1 | 0.61± 0.04* | 0.62 ± 0.07* |
| t_1/2_ (ms) | 10.8 ± 0.9 | 12.7± 0.9 | 13.9 ± 1.5 |
| Foot duration (ms) | 10.9 ± 0.6 | 15.9 ± 0.8* | 27.2 ± 2.9*^†^ |
| Foot amplitude (pA) | 8.1 ± 1.0 | 7.9 ± 0.6 | 7.9 ± 0.9 |
| Percentage of feet | 38.3 ± 2.9 | 37.6 ± 2.7 | 32.7 ± 4.1 |
| Number of cells | 22 | 20 | 16 |
