# Supplementary material for: The F-Actin Binding Protein Cortactin Regulates the Dynamics of the Exocytotic Fusion Pore through its SH3 Domain
Source: Front Cell Neurosci. 2017 May 4;11:130. doi: 10.3389/fncel.2017.00130 (PMC5415606; doi:10.3389/fncel.2017.00130)
Supplement: Supplementary file 5 [file Table_5.docx]

**Table S5**: *Amperometric parameters of exocytotic in cells expressing non-phosphorylatable cortactin mutants*. Chromaffin cells were transfected with cortactin wild-type (WT), non-phosphorylatable mutant S405,418A (2A) or non-phosphorylatable mutant Y421,466,482F (3F). 48 h later, exocytosis was induced with 50 µM DMPP and monitored by amperometry. Data are means ± SEM of median value determined for each cell. *p<0.05 compared with cells transfected with cortactin WT (ANOVA followed by unpaired t-test).

|  | WT | 2A | 3F |
| --- | --- | --- | --- |
| Number of events | 37.2 ± 3.2 | 20.7 ± 2.6* | 36.7 ± 5.8 |
| Q (pC) | 0.7 ± 0.1 | 0.8 ± 0.1 | 0.9 ± 0.1 |
| t_1/2_ (ms) | 11.4 ± 0.7 | 14.4 ± 1.2* | 14.8 ± 1.2* |
| Foot duration (ms) | 13.8 ± 1.1 | 17.1 ± 1.1* | 21.1 ± 2.2* |
| Foot amplitude (pA) | 7.2 ± 0.6 | 10.7 ± 1.0* | 9.5 ± 0.6* |
| Percentage of feet | 43.4± 3.4 | 47.5 ± 3.3 | 35.6 ± 3.0 |
| Number of cells | 34 | 33 | 32 |
